# Environmental Cues Facilitate Maturation and Patterning of Human Induced Pluripotent Stem Cell-Derived Cardiomyocytes

**DOI:** 10.33594/000000730

**Published:** 2024-10-04

**Authors:** Enrique Coca, Scott Cho, Christopher Kauffman, Alonzo D. Cook, Martin Tristani-Firouzi, Natalia S. Torres

**Affiliations:** a Nora Eccles Harrison Cardiovascular Research and Training Institute, University of Utah, Salt Lake City, Utah, USA; b InviRX Antiviral Therapeutics, Sandy, Utah, USA

**Keywords:** Human induced pluripotent stem cells, Cardiomyocytes, Extracellular matrix, Maturation

## Abstract

**Background/Aims:**

Advances in induced pluripotent stem cell (iPSC) technology allow for reprogramming of adult somatic cells into stem cells from which patient- and disease-specific cardiomyocytes (CMs) can be derived. Yet, the potential of iPSC technology to revolutionize cardiovascular research is limited, in part, by the embryonic nature of these cells. Here, we test the hypothesis that decellularized porcine left ventricular extracellular cardiac matrix (ECM) provides environmental cues that promote transcriptional maturation and patterning of iPSC-CMs in culture.

**Methods:**

Cardiac progenitor cells were plated on ECM or standard tissue plates (2D monolayer) for 30 days, after which CM orientation and single cell transcriptomics were evaluated using confocal imaging and singe cell RNA-sequencing, respectively.

**Results:**

Cardiac progenitors differentiated on left ventricular ECM formed longitudinal fibers that differed quantitatively from progenitors differentiated in standard 2D conditions. Unsupervised clustering of single cell transcriptomics identified a CM cluster expressing a higher level of genes related to CM maturation. CMs differentiated on ECM were overrepresented in this cluster, indicating a bias toward CM maturation, compared to cells differentiated in standard 2D monolayer conditions.

**Conclusion:**

Our data suggest that environmental cues related to the left ventricular ECM may promote differentiation to a more mature CM state compared to cells differentiated on a standard 2D monolayer, while facilitating organization into longitudinal micro-fibers. Our study highlights the utility of ECM as a differentiation substrate to promote CM maturation and fiber orientation *in vitro*.

## Introduction

Advances in induced pluripotent stem cell (iPSC) technology allow for reprogramming of adult somatic cells into stem cells from which patient-specific (and thus disease-specific) cardiomyocytes (CMs) can be derived. The impact of this technology has far-reaching implications, ranging from drug discovery [1], pre-clinical drug screening [2], mechanistic understanding of disease processes, and advances in personalized medicine. However, the potential of iPSC technology to revolutionize cardiovascular research and therapeutic development is limited by several key technological hurdles. For example, human iPSC-CMs in culture behave more like embryonic than mature myocytes and thus may not recapitulate key features of post-natal or adult-onset disease [3]. Multiple strategies have been employed to enhance maturation, including prolonged *in vitro* culture [4], electrical and mechanical stimuli [5, 6], co-culture strategies with non-cardiomyocytes [7–9], metabolic and hormonal modulation [10–12], and 3D culture strategies such as hydrogels [13] and cardiac organoids generation [14].

The cardiac extracellular matrix (ECM) is a complex 3D network composed principally of collagen fibers, but also including fibronectin, glycosaminoglycans and proteoglycans [15]. The ECM serves as a structural scaffolding for cells and structures that form the complex and intricate anatomic features of the mammalian heart. In theory, native ECM provides a relatively favorable microenvironment that might recapitulate cardiogenesis and promote maturation of cardiac cells *in vitro*. Indeed, several studies report improvements in iPSC-CM properties that reflect maturation when cells are plated with decellularized ECM preparations [16–18]. However, the molecular mechanisms whereby exposure to ECM promotes maturation remain unclear [3, 16]. Here, we test the hypothesis that decellularized porcine left ventricular ECM provides environmental cues that promote transcriptional maturation and patterning of iPSC-CMs in culture.

## Materials and Methods

### Human subjects

Human subject research for a blood draw and derivation of human iPSC was conducted under approval from the Institutional Review Board (IRB) at the University of Utah. Informed consent was obtained from research participants according to IRB guidelines.

### Extracellular Matrix Decellularization and Preparation

Decellularized ECM samples were obtained using a previously established protocol [19]. Briefly, juvenile female porcine hearts obtained from a slaughterhouse were rinsed in a PBS solution containing heparin and antibiotics, stored frozen in distilled water, then later thawed overnight. The hearts were decellularized using a retrograde Langendorff perfusion system by 3 alternating cycles of 0.5 % SDS and distilled water, one cycle of 1% Triton X-100 followed by a final rinse in distilled water. Portions of the left ventricular tissue were snap frozen and sectioned at 200 μm with a cryostat (Leica CM 1950, Leica Biosystem). From each section, 12 mm biopsies were cut, placed between round coverslips and sterilized in a 48-well plate with 65% EtOH, 1.92% Penicillin in distilled water for three hours. After three rinses with PBS (30 minutes each) the sections were stored in PBS at 4 °C until use.

### Sample Preparation for Scanning Electron Microscopy

Scanning Electron Microscopy (SEM) was used to visualize ECM ultrastructure. Decellularized ECM samples were fixed with 4% PFA for 1 hr, rinsed twice with 0.1 M sodium cacodylate for 10-minutes, and washed with 2% osmium tetraoxide in rinse buffer for 1 hr. This was followed by dehydratation using a critical point dryer with CO_2_ flushing followed by heating up to critical point. Samples were then mounted onto glass stubs using carbon adhesive, coated with gold and palladium and imaged in a Zeiss Gemini 300 at the University of Utah Electron Microscopy Core Laboratory.

### Human iPSC generation and cardiomyocyte differentiation protocols

**Fig. 1.**
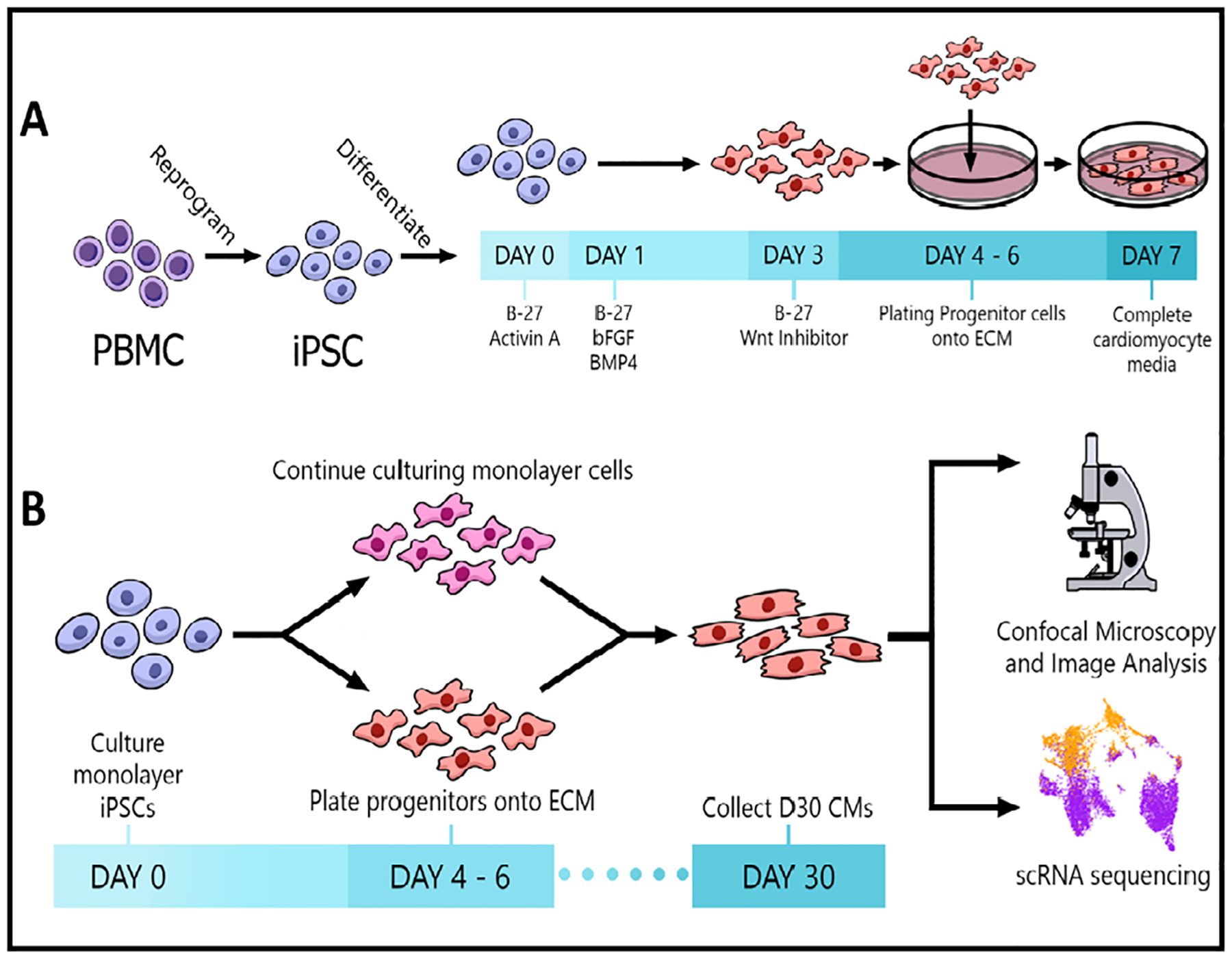
Experimental protocols. (A) Schematic of the iPSC reprogramming and CM differentiation protocol. (B) Plating strategy and data acquisition methods.

Peripheral blood mononuclear cells (PBMCs), isolated from human blood samples using Ficoll-Paque Plus (Fisher Scientific), were reprogrammed by sendai virus transfection of Yamanaka Factors: Oct 3/4, Klf-4, Sox2, and c-Myc according to the manufacturer’s feeder-free protocol (CTS CytoTune-iPS 2.1 Sendai Reprogramming Kit, Thermo Fisher Scientific) [20]. The differentiation and plating protocols for this study are depicted in Fig. 1. Human induced pluripotent stem cells (iPSC) were differentiated into cardiac progenitors (4–6 days) using the matrix-sandwich method, as previously described [20–24]. For standard 2D monolayer differentiation, cardiac progenitors were maintained in RPMI/B27/insulin media and allowed to spontaneously differentiate in this media from day 7 to day 30. For the ECM differentiation, cardiac progenitors were dissociated with TrypLE (Thermo Fisher Scientific), resuspended in RPMI/B27-no insulin media with Y-27632 (10uM, Selleckchem) and then plated (5 × 10^5^ cells) in FBS pre-coated ECM sections. On day 7, cells were fed with RPIM/B12/insulin media until day 30 when samples were collected.

### Immunohistochemistry and Confocal Microscopy

After a short rinse in phosphate buffered saline solution (PBS), ECM and 2D monolayers samples were fixed in 4% paraformaldehyde at room temperature for 10 minutes (2D monolayers) or 1 hr (ECM) on a rocker. After three rinses with PBS (30 min each) the samples were permeabilized (PBS-0.2% Triton X-100, 1 hr) and blocked (PBS-2% fetal bovine serum (FBS, Sigma Aldrich), 1hr) before an overnight incubation at 4 °C in blocking solution with primary antibodies: monoclonal anti-cardiac Troponin T (cTnT, RV-C2, 1:50, F5D; 1:50; Developmental Studies Hybridoma Bank and monoclonal anti-Vimentin (VIM, V6630, 1:200, Sigma Aldrich, St. Louis, MO). Following this incubation, the samples were washed three times with PBS for 30 minutes and incubated for 60 min with the appropriate secondary antibody: goat anti mouse IgG2b Alexa Fluor^®^ 647 (A21241, 1:500, Invitrogen, Eugene, OR) and goat anti mouse IgG1 Alexa Fluor^®^ 488 (A21121, 1:500, Invitrogen, Eugene, OR) diluted in incubation solution, at room temperature for one hour on a rocker. Following three PBS washes (30 min) the samples were mounted between two coverglass slips using FluoroMount-G Mounting Medium (Thermo Fisher Scientific). A two-track protocol was used to obtained 3D-stacks using a Zeiss LSM 880 Airyscan confocal microscope (Carl Zeiss, Jena, Germany) equipped with a 20x lens belonging to the Cell Imaging Core at the University of Utah. Images were processed using Imaris software (8.4.1 Bitplane AG, Zurich, Switzerland).

### Analysis of CM orientation

cTNT staining was used to analyze CM fiber directionality for each slice in confocal image stacks based on a previous published method [25] using the Directionality tool available in Fiji [26]. The combined histogram for each cultured condition was fitted with a Gaussian curve and the full width at half maximum (FWHM) was calculated using Origin(R). A zero degree angle indicates an align ment parallel with the main angle reported.

### Single Cell RNA sequencing

Single cells were dissociated from 2D monolayer and ECM samples using the STEMdiff Cardiomyocyte Dissociation Kit (STEMCELL) following manufacturer instructions. After resuspension in 0.04% FBA (Sigma Aldrich), barcoded single-cell 3’ cDNA libraries were generated using Chromium Single Cell 3’ gel bead and library Kit v3 (10x Genomics Pleasanton, USA) and sequenced using an Illumina NextSeq-500 at the University of Utah High-Throughput Genomics Core. Quality control of cDNA and final libraries was performed using Bioanalyzer High Sensitivity DNA Analysis (Agilent) or 4200 TapeStation System (Agilent). After sequencing, samples were mapped to the human reference genome GRCh37 using the CellRanger suite (10X Genomics) with default parameters. Cell Range outputs were then processed using the standard Seurat 3.1 package workflow in R [27] to perform data analysis and quality control. Cells were clustered as CM or fibroblast based on their expression of key genes: TNNT2, RYR2, TNNI1 and MYH6 for CMs; VIM, COL1A1, COL1A2 and DDR2 for fibroblasts. Any cells expressing both fibroblast and CM genes were labeled as combined expression and not included in subsequent analysis. Differential gene expression among cell types and clusters was calculated by Seurat, and pathways analysis was done by DAVID [28, 29]. We performed sub-group analysis within the CM cluster to futher characterize each culture condition.

### Statistics

All data were presented as means ± SE. For each data set, n indicates the number of individual differentiations in a given condition. Statistical analysis was performed using the Kolmogorov-Smirnov Test to compare sample distributions, a two-tailed Student’s t test when comparing two groups, and the Chisquared test when analyzing categorical data. Precise P values were reported when possible, with a P < 0.05 considered to be significant.

## Results

### Scanning electron microscopy of decellularized ECM revealed a porous collagenous structure

**Fig. 2.**
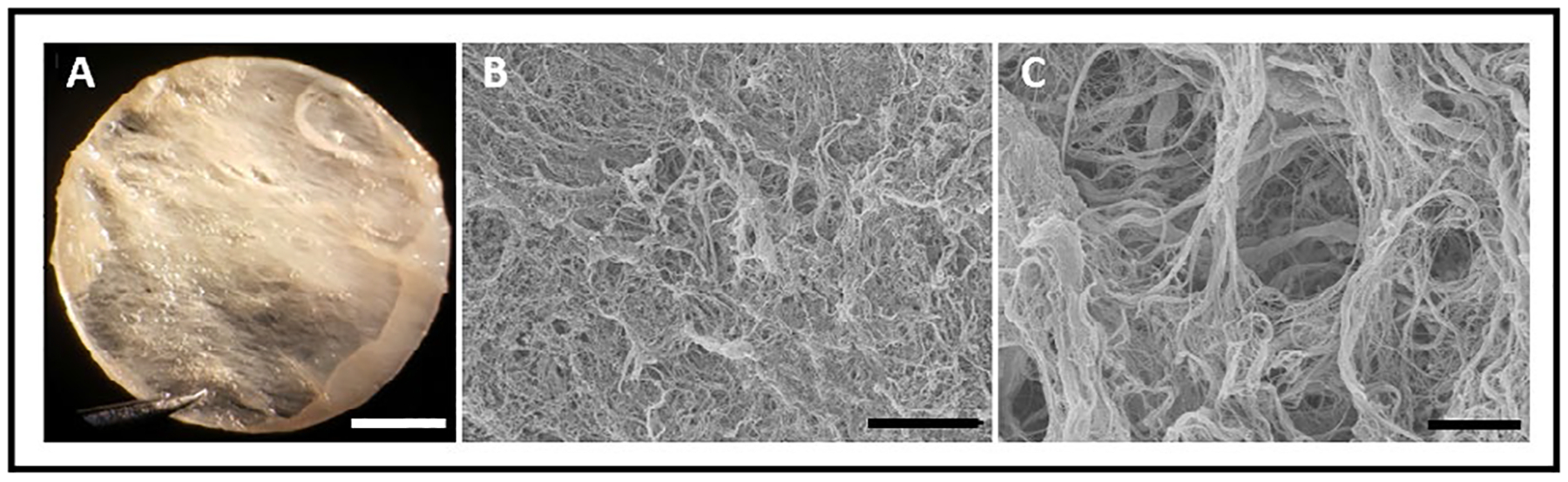
Macro- and micro-structural features of the left ventricular extracellular matrix (ECM). (A) Light microscopy image showing ECM macrostructure after decellularization. The resulting membrane is translucent and comprised of large fibers. (B, C.) ECM microstructure by SEM reveals the collagen fibers and tunnels that offer support to CMs differentiation and development. Scale bars 2 mm(A), 75 μm (B) and 10 μm (C).

Scanning Electron Microscopy (SEM) was used to study the micro-structure of the decellularized ECM preparation. The resulted images revealed that the translucid ECM (Fig. 2A) was composed of a complex network of interspersed fibrils presenting a honeycomb structure similar to human left ventricle [30] (Fig. 2B). Higher amplification reveled the presence of perimysium (collagenous connective tissue that surrounds fibers), creating entry points for the cells within the three-dimensional structure of the substrate.

### Cardiac progenitors plated on porcine ECM formed longitudinal strands of CMs and fibroblasts

**Fig. 3.**
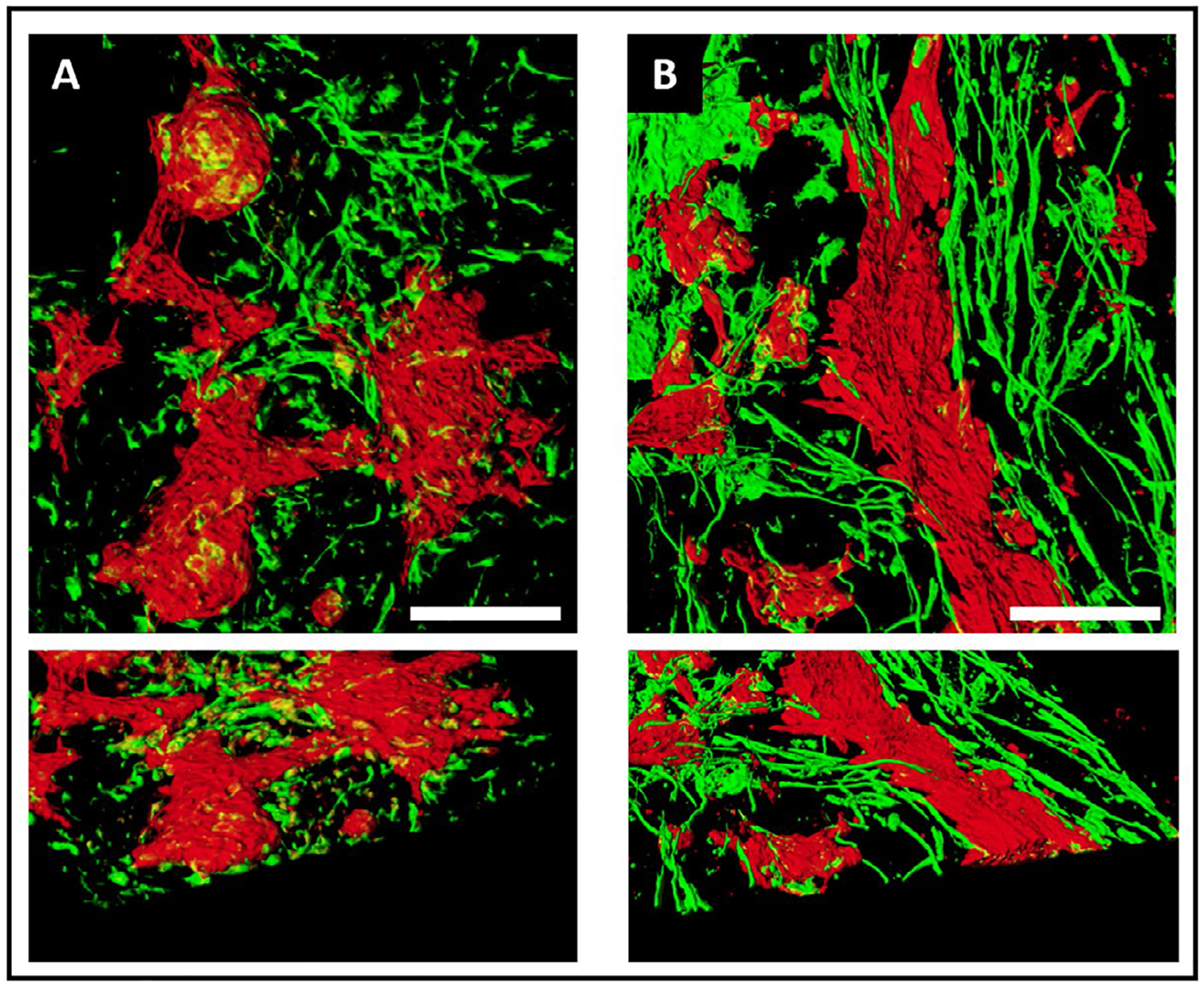
Cardiac progenitors plated on left ventricular ECM formed longitudinal strands of CMs and fibroblasts. Top views of 3D reconstructions of 2D monolayers (A) or ECM (B) samples. iPSC-CMs (cTNT, red) differentiated on standard tissue culture plates formed clusters of cells that interconnected via strands, with fibroblasts (Vimentin, green) lacking orientation. In contrast, in ECM samples CMs formed longitudinal fibers with adjacent stands of fibroblasts. Lower panels show a 45° perspective view of the same samples highlighting the thickness and fibers in the ECM sample. Scale bar = 100μm.

To evaluate if the differentiation micro-enviroment impacted cell development or organization, we seeded cardiac progenitor cells on standard tissue culture plates (2D monolayer) or ECM preparations and compared the study conditions after 30 days of differentiation. 2D monolayer and ECM samples were immunostained using antibodies to TNNT2 and VIM, to demarcate CMs and fibroblasts, respectively. Confocal microscopy revealed distinctive patterning of cells differentiated in the two conditions. By visual inspection, the overwhelming majority of ECM-plated CMs formed longitudinal fibers across the imaging plane (~400 uM), alongside fibroblasts (Fig. 3). By contrast, in the 2D monolayer condition, CMs and fibroblasts showed no apparent directionality. Moreover, 2D monolayer samples displayed a thickness resemblant of a single-cell layer [31] while ECM-plated samples showed multicellular fibers present throughout various depths of the imaged sample (2D monolayer thickness = 18.4 ± 2.5 μm (n=5) vs. ECM thickness = 49.1 ± 9.0 μm (n = 8); p = 0.008).

### ECM environment favored alignment of CM fibers

**Fig. 4.**
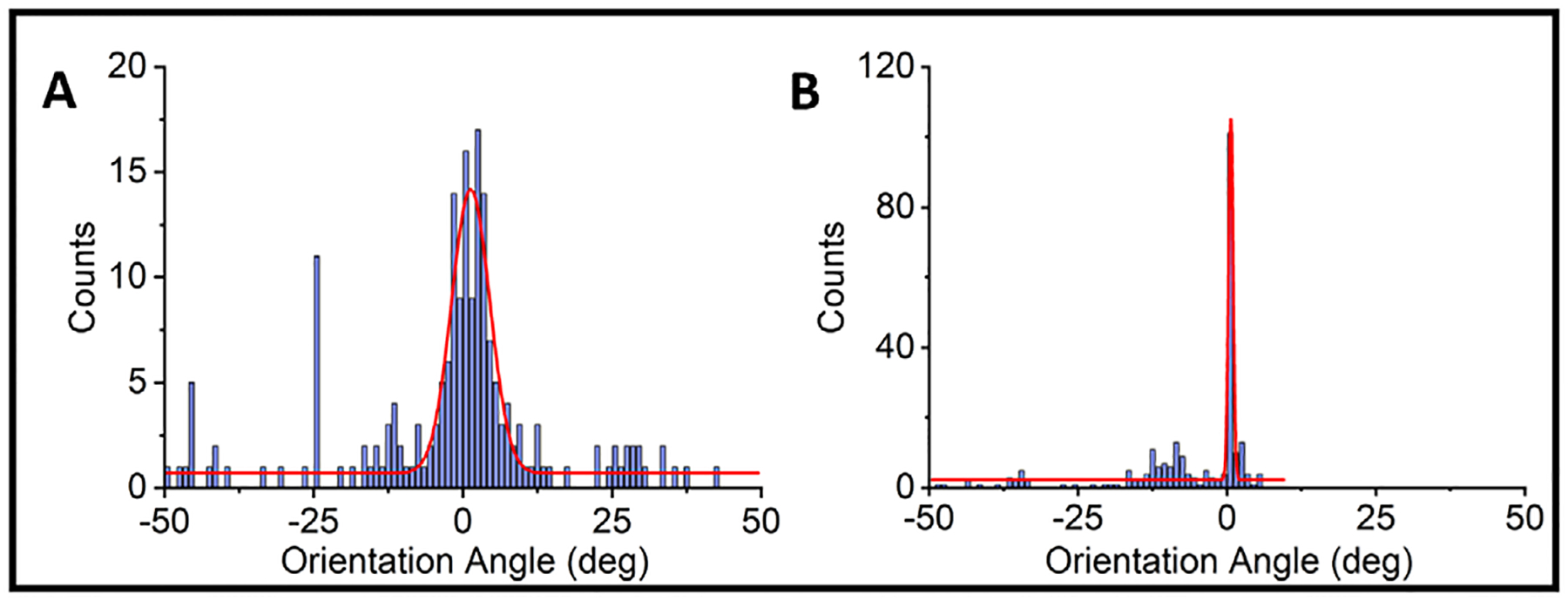
Quantification of linear strands using measurements of orientation angles. The orientation of CMs for each image of the confocal z-stack was measured using the cTNT signal. Orientation angle of 0 degrees represents a straight line. Deviation from a straight line is represented by orientation angles in the positive and negative directions. Orientation angles of 2D monolayer CMs (A) were widely scattered, indicating the absence of a specific linearity across the image. N = 9 monolayer preparations and 210 z-stack slices. By contrast, the orientation angles of CMs differentiated on the ECM (B) were highly centered on 0 degrees, indicating a linear orientation of fibers across the ECM (N = 7 ECM preparations and 240 z-stack slices).

Visual inspection of ECM plated cardiomyocytes (as identifed by cTNT+ staining) revealed organization into longitudinal fibers, as compared to the 2D monolayers (Fig. 3). To quantify the degree of longitudinal orientation, the major axis of CM orientation was measured using the cTNT signal and an alignment angle was calculated with respect to the major direction of the signal. The alignment angles across all CMs on each confocal z-stack for both ECM-plated and 2D monolayers were compiled into a histogram in 10-degree bins (Fig. 4). A zero-degree angle indicates that CMs were aligned parallel along a longitudinal pattern. The orientation angles of CMs differentiated on the ECM were highly centered on 0 degrees, indicating a linear orientation of fibers across the ECM. By contrast, the orientation angles of 2D monolayer CMs were widely scattered, indicating the absence of a specific linearity across the image. To further quantify the differences in orientation angles, the ECM-plated and 2D monolayer distributions were fitted with a Gaussian function and the Full Width at Half Maximum (FWHM), a measure of dispersion of the Gaussian function, was calculated. The FWHM for the ECM samples was 0.91° ± 0.13 (n=244), compared to 7.56° ±0.51 (n=207) for the 2D monolayer differentiations (Kolmogorov-Smirnov Test, p < 0.0001). This analysis confirmed our visual observation that CMs differentiated on the ECM indeed formed longitudinal strands, suggesting that progenitor cells utilized microstructural environmental cues to facilitate formation of fibers, much akin to the developing human heart. Images from all ECM samples can be found in Supplemental Fig. 1.

### Transcriptional features of ECM-differentiated cardiomyocytes displayed a more mature profile

**Fig. 5.**
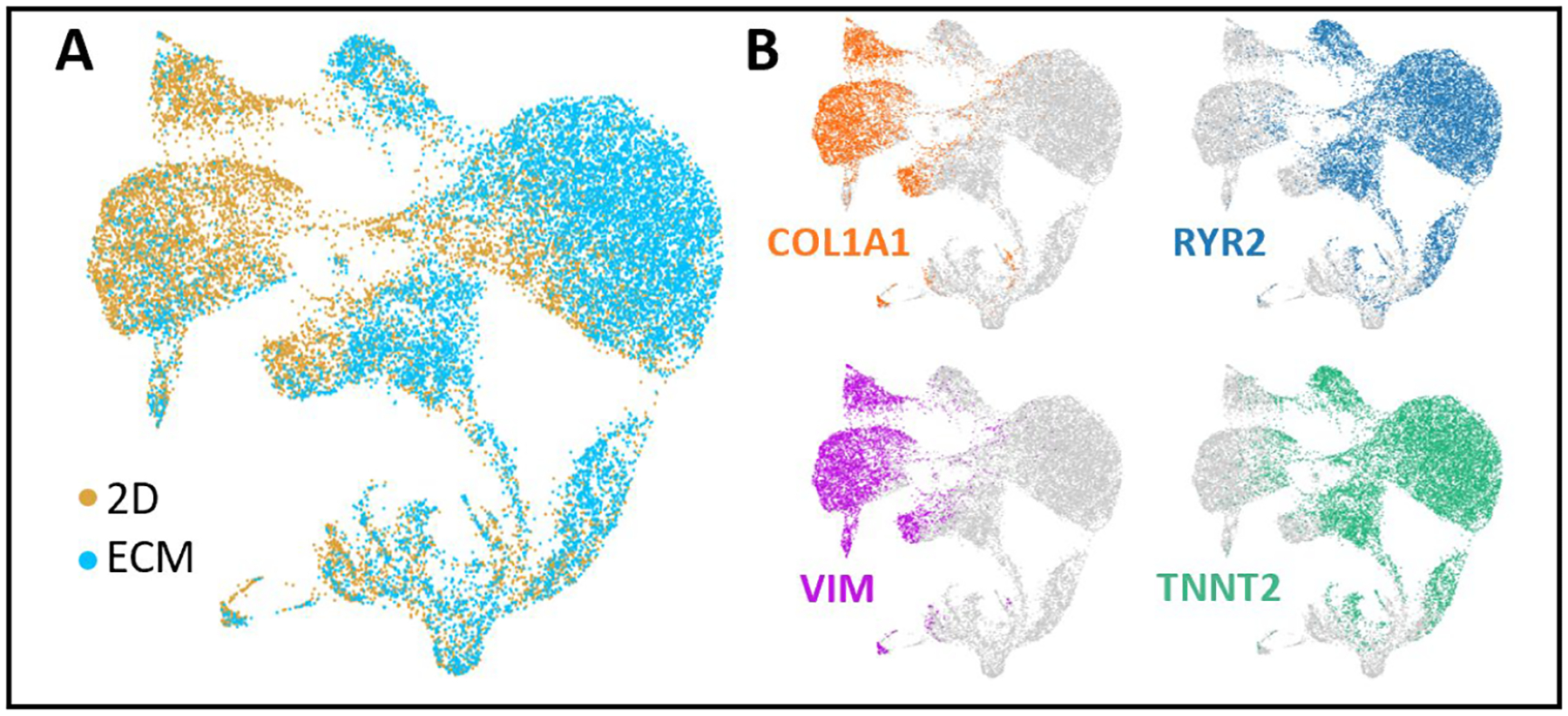
Single cell transcriptome of 2D and ECM samples. t-distributed stochastic neighbor embedding (tSNE) plots of combined 2D and ECM cultured cells. Each dot represents a single cell labelled according to culture condition (A) or cell type (B), with COL1A1 and VIM labelling fibroblasts and RYR2 and TNNT2 labelling CMs.

Next, we characterized the molecular properties of cells differentiated in the ECM versus 2D monolayer, using single-cell RNA-Seq. Fig. 5 summarizes the single cell RNA-Seq data derived from the 2 differentiation protocols and analyzed using unsupervised clustering algorithms. CMs and fibroblasts were readily distinguished by RYR2 and TNNT2, and COL1A and VIM expression, respectively. Moreover, K-means clustering based on gene expression using Gene Ontology (GO) terms revealed 2 distinct categories of cells: a cluster expressing gene pathways related to cardiac muscle contraction, ventricular morphogenesis, sarcomere organization (CMs); and a cluster enriched in gene pathways related to extracellular matrix, extracellular space, basement membrane (fibroblasts) (Table 1). Thus, single cell RNA-SEQ allowed us to distinguish specific sub-populations of cells, in this case, CMs and fibroblasts, based on gene expression. A very small proportion of cells (<5%) expressed the endothelial marker PECAM1 (not shown).

**Fig. 6.**
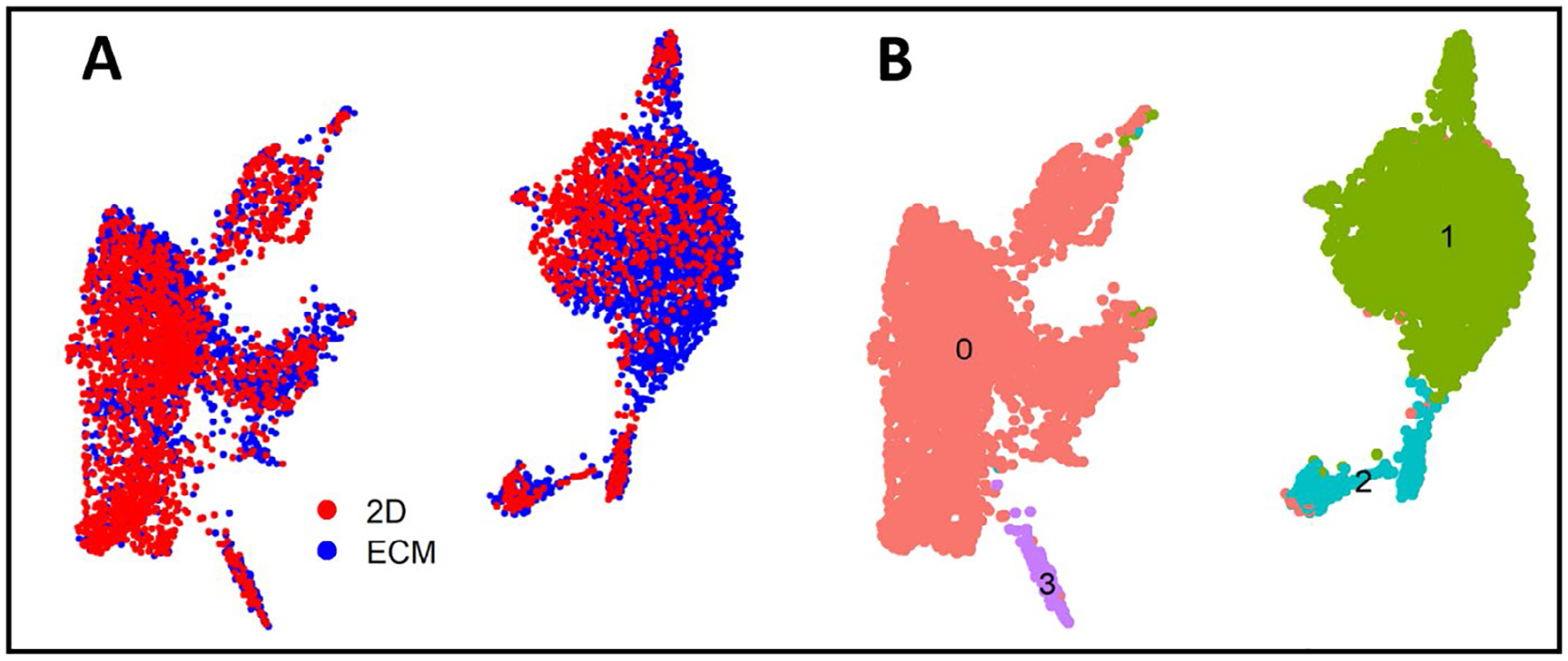
CMs differentiated on left ventricular ECM were enriched for genes related to cardiac development and contraction. Single cell RNA-Seq analyses comparing the CM transcriptome (as defined by RYR2 expression) for 2D monolayer and ECM differentiation protocols. (A) t-SNE plot of single CM cell transcriptomes for 2D monolayer (red) and ECM differentiation protocol (blue). (B) The same t-SNE plot as in (A), using unsupervised clustering to identify 4 unique clusters (0–3).

To identify changes in the cardiomyocyte transcriptome associated with culture conditions, we selected all CMs (defined by RYR2 expression) and re-analyzed the data using unsupervised clustering to define 4 clusters of cells. As 90% of the cells mapped to cluster 0 or 1, we restricted our analysis to these clusters (Fig. 6). One of the major clusters (cluster 0) contained equal numbers of ECM and 2D monolayer derived cells. However, cluster 1 contained an over-representation of ECM cells as compared to Cluster 0 (chi-squared, p-value < 2.2 E-16). While both clusters represent CMs (based on RYR2 expression), cluster 1 expressed greater levels of genes related to cardiac development and maturation, while cluster 0 expressed gene pathways related to earlier CM development, such as Wnt signaling (Table 2). These data suggest that environmental cues related to the ECM cells may promote differentiation to a more mature CM state compared to cells differentiated on the 2D monolayer.

## Discussion

Human iPSC-CMs have emerged as an informative platform for the study of cardiac pathophysiology, drug discovery [1], and pre-clinical drug screening [2], in addition to promoting mechanistic insights into cardiovascular disease. Yet, the potential of iPSC technology to revolutionize cardiovascular research and therapeutic development is limited, in part, by the embryonic nature of these cells [3]. Here, we show that using porcine left ventricular ECM as a differentiation environment promotes unique patterning and transcriptional maturation of the resulting iPSC-CMs.

The cardiac ECM is a complex 3D network composed principally of collagen fibers, but also including fibronectin, glycosaminoglycans and proteoglycans at various levels [15]. Our SEM images highlight the microstructural intricacies of the matrix, with collagen fibers surrounding tunnels that offer support for CM differentiation and development. Thus, the ECM provides a structural scaffold for cells that form the complex and intricate anatomic features of the mammalian heart. We wondered if the ECM scaffold might provide environmental cues that promote patterning and maturation of iPSC-CMs.

In this context, we observed that cardiac progenitors differentiated on the porcine left ventricular ECM formed longitudinal fibers, along with fibroblasts, that differed qualitatively and quantitatively from progenitors differentiated within standard 2D conditions. iPSC-CMs differentiated under standard 2D conditions tended to form balls or clusters of cells, without any clear longitudinal patterning. Indeed, the orientation angles of 2D monolayer CMs were widely scattered, indicating the absence of a specific linearity across the confocal image. By contrast, the orientation angles of CMs differentiated on the ECM were highly centered on 0 degrees, indicating a linear orientation of fibers across the ECM. Taken together, these observations indicate that as a differentiation substrate, left ventricular ECM promoted the organization of CMs along longitudinally oriented micro-fibers.

Unsupervised clustering of single cell transcriptomics allowed us to identify sub-populations of CMs that were uniquely enriched in cells differentiated in the ECM environment versus the standard 2D monolayer. The majority of CMs localized to 2 clusters based on gene expression profiles. One of the clusters (Cluster 0) was enriched for terms related to early CM development, such as Wnt signaling. There was equal representation of ECM and 2D monolayer differentiated CMs in this cluster. By contrast, Cluster 1 expressed a higher level of genes within GO terms related to CM maturation. CMs differentiated on ECM were overrepresented in this cluster. These findings suggest that the CMs differentiated on the left ventricular ECM were more biased toward a population of more mature cells, compared to cells differentiated in standard 2D monolayer conditions.

There are several limitations inherent in this study. We focused exclusively on the impact of two distinct differentiation substrates, the left ventricular ECM and the standard 2D monolayer, without consideration of additional environmental cues such as mechanical stretch or electrical stimulation. It is plausible that these interventions, when coupled with the ECM environmental cues, might further enhance CM maturation and orientation. Additionally, CM differentiation protocols that facilitate formation of cardiac organoids also hold tremendous promise for 3D organization that may promote maturation [14].

## Conclusion

Our data suggest that environmental cues related to the left ventricular ECM may promote differentiation to a more mature CM state compared to cells differentiated on the 2D monolayer, while facilitating organization into longitudinal micro-fibers. These findings lay the foundation for future studies to investigate how specific cues provided by the ECM environment activate the transcriptomic pathways identified here. Our study highlights the utility of ECM as a differentiation substrate to promote CM maturation and fiber orientation *in vitro*.

## Supplementary Material

Suppl Material

## Figures and Tables

**Table 1.** Upregulated GO terms in cell populations

GO Term	P-Value
Cardiomyocytes	
Cardiac muscle contraction	1.50E-15
Muscle filament sliding	1.60E-15
Regulation of cardiac conduction	4.70E-10
Sarcomere organization	6.40E-07
Cardiac muscle fiber development	1.00E-05
Striated muscle contraction	4.60E-04
Response to muscle stretch	1.70E-02
Fibroblast	
Extracellular matrix organization	6.80E-04
Collagen fibril organization	7.90E-04
Collagen catabolic process	6.00E-03
Actin crosslink formation	2.50E-02
Extracellular fibril organization	3.10E-02
Actin filament organization	4.2 0E-02

**Table 2.** Upregulated GO Terms in CM subcluster analysis. While both Cluster 0 and 1 in Figure 6B represent CMs, Cluster 1 expressed a higher level of genes within gene ontology (GO) terms related to CM maturation, while Cluster 0 was enriched for terms related to early CM development, such as Wnt signalling. CMs differentiated on ECM were overrepresented in Cluster 1, that reflects a more mature CM transcriptome

GO Term	P-Value
Cluster 0	
Wnt signaling pathway	8.60E-26
Tumor necrosis factor-mediated signaling pathway	2.60E-17
Proteolysis involved in cellular protein catabolic process	7.20E-05
Muscle contraction	4.50E-03
Negative regulation of apoptotic process	1.80E-02
Cluster 1	
Cardiac muscle contraction	2.90E-05
Cardiac muscle cell differentiation	7.50E-05
Cardiac muscle fiber development	2.60E-04
Sarcomere organization	3.60E-03
Cardiac myofibril assembly	2.60E-02
Regulation of the force of heart contraction	3.50E-02
