# Supplementary material for: Environmental Cues Facilitate Maturation and Patterning of Human Induced Pluripotent Stem Cell-Derived Cardiomyocytes
Source: Cell Physiol Biochem. Author manuscript; Available in PMC 2025 Mar 31. (PMC11957376; doi:10.33594/000000730)
Supplement: Suppl Material [file NIHMS2068872-supplement-Suppl_Material.pdf]

# Supplementary Material

# Environmental Cues Facilitate Maturation and Pat terning of Human Induced Pluripotent Stem Cell-Derived Cardiomyocytes

Enrique Coca[a] Scott Cho[a] Christopher Kauffman[a] Alonzo D. Cook[b]
Martin Tristani-Firouzi[a] Natalia S. Torres[a]

[a]Nora Eccles Harrison Cardiovascular Research and Training Institute, University of Utah, Salt Lake City, Utah, USA,
[b]InviRX Antiviral Therapeutics, Sandy, Utah, USA

Supplemental Figure 1

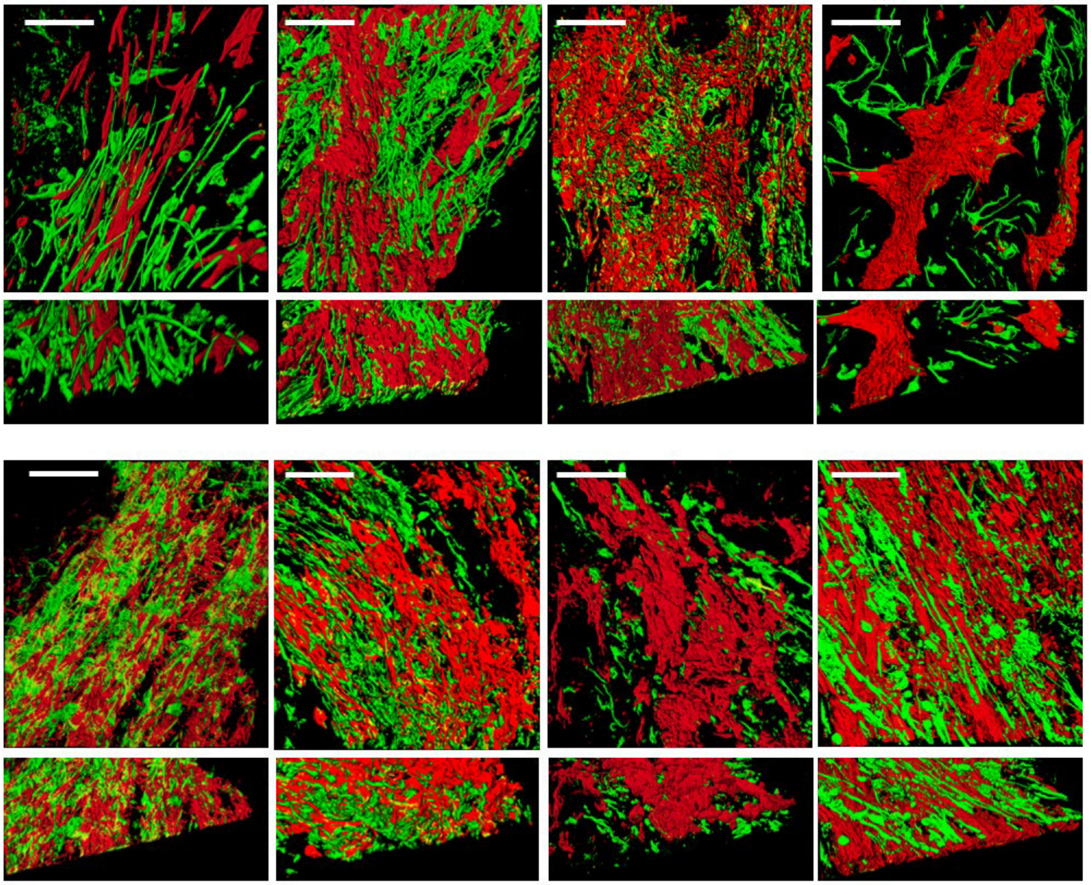

Supp. Fig. 1. Series of ECM 3D reconstructions. Eight samples differentiated on left ventricle ECM showing CMs (cTNT, red) and Fibroblast (Vimentin, green) orientations. While there is variation among the ECM samples, the alignment is higher than what is typically found in samples culture in 2D monolayers (see Figure 3A). Lower panels show a 45° perspective view of the same samples. Each example below represents a unique ECM preparation. Scale bar = 100μm.
